# Phase-amplitude cross-frequency coupling in the human nucleus accumbens tracks action monitoring during cognitive control

**DOI:** 10.3389/fnhum.2013.00635

**Published:** 2013-10-07

**Authors:** Stefan Dürschmid, Tino Zaehle, Klaus Kopitzki, Jürgen Voges, Friedhelm C. Schmitt, Hans-Jochen Heinze, Robert T. Knight, Hermann Hinrichs

**Affiliations:** ^1^Departments of Neurology and Stereotactic Neurosurgery, Otto-von-Guericke UniversityMagdeburg, Germany; ^2^Department of Behavioural Neurology, Leibniz Institute for NeurobiologyMagdeburg, Germany; ^3^German Center for Neurodegenerative Disease (DZNE)Magdeburg, Germany; ^4^Department of Psychology, Helen Wills Neuroscience Institute, University of CaliforniaBerkeley, Berkeley, CA, USA; ^5^Department of Psychology, University of CaliforniaBerkeley, Berkeley, CA, USA

**Keywords:** phase-amplitude coupling, nucleus accumbens, cognitive control, action monitoring, learning

## Abstract

The Nucleus Accumbens (NAcc) is an important structure for the transfer of information between cortical and subcortical structures, especially the prefrontal cortex and the hippocampus. However, the mechanism that allows the NAcc to achieve this integration is not well understood. Phase-amplitude cross-frequency coupling (PAC) of oscillations in different frequency bands has been proposed as an effective mechanism to form functional networks to optimize transfer and integration of information. Here we assess PAC between theta and high gamma oscillations as a potential mechanism that facilitates motor adaptation. To address this issue we recorded intracranial field potentials directly from the bilateral human NAcc in three patients while they performed a motor learning task that varied in the level of cognitive control needed to perform the task. As in rodents, PAC was observable in the human NAcc, transiently occurring contralateral to a movement following the motor response. Importantly, PAC correlated with the level of cognitive control needed to monitor the action performed. This functional relation indicates that the NAcc is engaged in action monitoring and supports the evaluation of motor programs during adaptive behavior by means of PAC.

## 1. Introduction

The nucleus accumbens (NAcc) is part of the ventral striatum and plays a pivotal role in integration of information (Goto et al., 2008) from the limbic system, particularly between the prefrontal cortex (PFC) and the hippocampus (HC). The NAcc is considered the interface by which the HC gates input from the prefrontal cortex (French et al., 2002). In rats the PFC and HC converge onto single NAcc-neurons (Finch et al., 1996; Goto et al., 2008) and the PFC-NAcc and HC-NAcc connections are mutually dependent. For instance, long term potentiation of the HC-NAcc association entails a long term depression of the PFC-NAcc association (Grace et al., 2007). It is assumed that this selective strengthening of the HC-NAcc connection is important for the rapid facilitation of goal-directed behaviors and for supporting automized actions (Goto et al., 2005; Belujon et al., 2008). Such automized actions are especially evident in motor learning tasks in which the NAcc integrates information for the planning of movements (Mogenson et al., 1980; Grace et al., 2000). (Münte et al. 2007) speculated that the human NAcc evaluates the information used for the adjustment of response strategies. Accordingly, lesions in the NAcc limit the flexibility required for changes in behavior during learning (Grace et al., 2007). However, knowledge about the specific neural mechanisms utilized to integrate information from the PFC and the HC in the human brain is still limited.

Phase-amplitude cross-frequency coupling (PAC) of oscillations has been suggested as an effective mechanism for recruiting local networks to form functional global networks and to gate information (Buzsaki et al., 2004; Canolty et al., 2006, 2010; Cohen et al., 2009; Staudigl et al., 2012). PAC describes the dependency of the amplitude of a high frequency on the phase of a low frequency. In rats and mice there is a tight connection between the phase of the theta band (θ) of local field potentials (LFP) and single unit activity (SUA) (Chrobak et al., 1998; Sirota et al., 2003; Siapas et al., 2005) presumably allowing neurons to form a larger assembly of neurons by means of transient coupling (Chrobak et al., 1998). These studies suggest that the interaction between PFC and HC may occur via PAC. O'Donnell et al. (1995) showed that hippocampal hyper- and depolarization leads to hyper- and depolarization in the NAcc. In the state of depolarization neurons in the NAcc are more likely to fire action potentials in response to stimulation of the PFC (French et al., 2002; Goto et al., 2008) providing evidence for PAC with an enhancement of high frequency amplitudes during troughs in θ activity. Tort et al., (2008) showed transient θ phase—high gamma (γ) coupling in the rat's striatum during movement through a maze. Furthermore, Tort et al. (2009) demonstrated a function link between performance improvement and the strength of theta-gamma coupling during the course of learning. However, until now it has not been established whether NAcc shows PAC between θ and high γ activity in a functionally specific manner in humans. This would indicate that integration of information within the NAcc could rely on transient coupling between frequencies.

We studied the NAcc activity in three human subjects directly by means of subcortical electrodes in a serial reaction time task (Nissen et al., 1987) in which the participants had to track and respond to a sequence of numeric stimuli in a fixed and a random order condition which modulated different cognitive control demands. From Tort et al. (2009) one can hypothesize that PAC transiently occurs in the NAcc and is modulated by the level of cognitive control. We tested the following hypotheses: PAC emerges in the NAcc. Second, PAC discriminates between phases of high and low cognitive control. Third, PAC varies systematically with behavioral performance measures across experimental conditions (high and low cognitive control—HCC and LCC).

## 2. Materials and methods

### 2.1. Participants

3 patients (mean age 38.3 years (*SD* = 12.34), 2 female, all right handed) with a history of intractable epilepsy participated in this study. We directly recorded from the bilateral NAcc and the anterior Thalamus (ANT). For details on surgery and deep brain stimulation approach please see Appendix 6.1 and Table A1 summarizing the clinical background of the patients.

### 2.2. Paradigm

**Figure 1:**
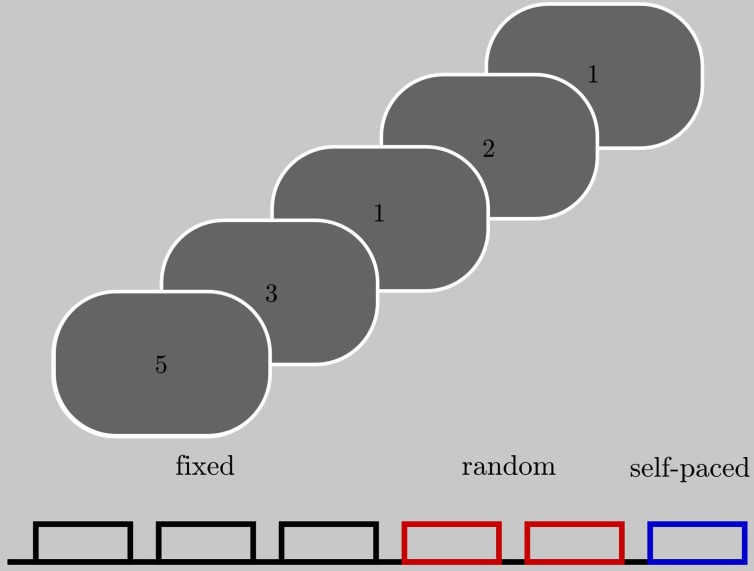
**Subjects were presented with stimuli indicating which finger to move**. Each block contained 60 trials (presentation of a single number). Colors denote the type of the sequence. Together, the subjects were presented with three blocks of a fixed sequence, two blocks of a random sequence, and one block in which they could choose a sequence on their own.

We carried out a serial reaction time task (see Figure 1) which required a single finger movement that was specified by a numeric stimulus presented on a monitor screen. Stimulus presentations were controlled by Matlab. Patients were instructed to respond with their right thumb, index finger, middle finger, or little finger, which rested respectively on the spacebar, “j,” “k,” and “;” keys of a computer keyboard. Four different numbers (1, 2, 3, 5) were presented on the screen (height 2 cm, 0.15° visual angle). These numbers indicated the finger they had to use to press the key. Six blocks of 60 trials, each comprising the presented number and the corresponding finger movement, were conducted with each patient. The four numbers were presented in a fixed or random order depending on the block number, with 3 fixed order blocks followed by 2 random sequence blocks and a final block of self-paced finger movements. In the fixed order blocks a repetitive sequence of six numbers was presented in all three blocks. In sum, the participant performed 30 repetitions of the 6-number-sequence. In the random blocks the four numbers were presented randomly. In the self-paced block a fixation cross was presented instead of the numbers. The participants were not informed about the type of sequence. The interstimulus interval (ISI) was variable and depended on the reaction time of the participants, with a fixed time between response and next stimulus of 700 ms plus a jitter of ±110 ms. In this interval the stimulus remained presented. Thus, block and trial length depended on the participants reaction time (mean block length per participant: Pat01: 117 s (std: 18.4), Pat02: 93 s (std: 25.6), Pat03: 96 s (std: 26.03); mean trial length per participant: Pat01: 1.6 s (std: 0.52), Pat02: 1.01 s (std: 0.31), Pat03:1.03 s (std: 0.21). Blocks were separated by a 1 min rest. During this resting period an X was presented on the screen. A + presented for 5 s informed the participant about the beginning of a new block.

### 2.3. Cognitive control

These 3 types of sequences (fixed, random and self-paced) differed with respect to the need for ongoing monitoring of actions and performance outcomes and subsequently, adjustments of behavior and learning which we tagged cognitive control in accordance to MacDonald et al. (2000) and Ridderinkhof et al. (2004).

#### 2.3.1. Fixed sequence

The fixed sequence allowed the participant to learn the sequence. During the early phase (cognitive phase Fitts et al., 1973) of learning, when the fixed sequence is unknown, a high level of cognitive control is necessary to establish a strategy to complete the task (Fitts et al., 1973) namely to associate the stimulus with the response. The longer the training the less necessary cognitive control is, since the stimulus-response association was learned and the participant knows which finger to move before the actual stimulus is presented on the screen.

#### 2.3.2. Random sequence

The sudden onset of a novel or unpredictable event captures attention and disrupts ongoing performance (Barcelo et al., 2006). In our experiment switching from the fixed to the random sequence marks such onset of several unpredictable events. This interruption of stimulus predictability signals the need for a change in strategy from a learned and hence, automatic to an unlearned mode. The occurrence of errors makes cognitive control necessary which leads to post-error slowing in healthy participants and more careful responses after errors (Notebaert et al., 2009). Hence, less errors are expected during phases of high cognitive control. In healthy participants, differences in cognitive control are reflected by a reduction of initially long reaction times after the completion of several repitions of the 6-item sequence of the same fixed sequence (Nissen et al., 1987; Knopman et al., 1991). The presentation of random sequences after the fixed sequences will force the participant to abandon a learning strategy and elevate reaction times to a plateau value.

#### 2.3.3. Self-paced sequence

During the last block, the participants could chose the sequence on their own with no obligation. Since the selected movement had not to be adjusted according to an external stimulus we expected short reaction times which would indicate less action monitoring or cognitive control. In sum, we classified trials of high cognitive control (HCC - initial tracking of the fixed sequence and tracking the random sequence) and low cognitive control (LCC - tracking the learned fixed sequence and during the self-paced sequence).

### 2.4. Data collection

Intracranial recordings were obtained using a Walter Graphtek (Walter Graphtek GmbH, Lübeck, Germany) system, with a sampling rate of 512 Hz, a resolution of 0.25 μV, and analog bandwidth of 200 Hz. We referenced online to the right earlobe. The ground electrode was placed at P8. During recording, a highpass filter of 0.19 Hz and a lowpass filter of 240 Hz was used. In the left and right NAcc and ANT (a total of 16 recording electrodes), adjacent electrodes were combined with each electrode referenced to the neighboring contact (i.e., 1–2, 2–3, 3–4, with “1” representing the most ventral and “4” representing the most dorsal electrode contact). This resulted in a bipolar montage with each NAcc/ANT monitored by three electrode positions. This montage was used to enhance the spatial resolution of the intracranial recordings and to ensure that the recorded activity originated from nearby tissue.

### 2.5. General data analysis

We used Matlab 2008a (Mathworks, Natick, USA) for all offline data processing. The resulting time series for the electrodes located in the NAcc were segmented in epochs of −1 to 2 s relative to the event (stimulus and response). In separate analyses these epochs were aligned to the motor response or onset of the instructive stimulus. These time series were used to characterize event-related brain dynamics in terms of PAC. We inspected the signal visually for artifacts and decided not to reject any epochs. Since we focused on single frequency bands we avoided signal drifts by applying bandpass filters for the frequency bands of interest (see below). All filtering was done using a 4th order butterworth filter (IIR-filter). All steps of data analysis were applied also to the recordings from the anterior thalamus.

### 2.6. Behavioral data

Two behavioral parameters—reaction times (RT) and error rate (*p*_*e*_)—were assessed as indicators of cognitive control. The DBS procedure allows for the recording of only a limited pool of subjects (here *N* = 3). This limited number of subjects may influence strongly statistical test results so that effects can be missed even though potentially observable in a larger set of subjects. An ANOVA comparing RT differences across blocks—which may indicate differences in cognitive control—therefore, used RT of each trial in each subject as the random variable (*n* = 180) and blocks as factors (*p* = 6). Reaction times of each subject were *z*-scored across trials. Individual reaction times are summarized in Table 1. The summed number of errors in each participant was used to calculate *p*_*e*_ for each block except the self-paced block (errors cannot be made since the sequence was generated by the participant itself) we calculated

(1) $$p_{e}=\frac{N_{\text{errors}}}{N_{\text{trials}}}$$

**Table 1A:** **Reaction times in ms**.

**Patient**	**Block # 1**	**Block # 2**	**Block # 3**	**Block # 4**	**Block # 5**	**Block # 6**
1	1027 (301)	1090 (261)	1077 (255)	1108 (284)	1057 (212)	645 (395)
*z*-score	0.079 (0.934)	0.268 (0.786)	0.229 (0.769)	0.323 (0.858)	0.170 (0.640)	−1.071 (1.191)
2	881 (214)	658 (106)	628 (146)	639 (122)	721 (180)	210 (182)
*z*-score	0.991 (0.823)	0.135 (0.407)	0.022 (0.562)	0.063 (0.469)	0.377 (0.692)	−1.588 (0.700)
3	814 (290)	642 (158)	604 (148)	655 (116)	680 (135)	510 (191)
*z*-score	0.804 (1.431)	−0.045 (0.782)	−0.230 (0.733)	0.022 (0.574)	0.143 (0.667)	−0.695 (0.943)

The table shows the mean reaction times and standard deviations of reaction times for each subject and block. Each value encompasses 60 trials. Since subjects differ in their reaction times (see Results) we *z*-scored the reaction times across all trials for each subject. This means that individual RTs were transferred into standard values by z-transformation individually for each subject. The second row of each patient shows the mean (std) of the *z*-scored reaction times. The subjects showed a different evolution of RTs across the experiment. But this concomitant in each group analysis—that individual subjects show only a trend which resembles the result of the group analysis or may slightly deviate from the trend in the group—does not challenge the statement resulting from the ANOVA for a group of participants. Even though we could only dispose of a very small group, the trend of behavioral changes in terms of RTs remains.

**Table 1B:** **Error rate**.

**Patient**	**Block # 1**	**Block # 2**	**Block # 3**	**Block # 4**	**Block # 5**
1	4	6	7	5	1
2	1	1	2	4	1
3	3	2	1	0	2

Table shows the number of errors for each subject and block.

with N_errors_ designating the set of trials with false responses and *N*_trials_ as the total number of trials in a given block. A χ^2^ test statistically compared the blockwise *p*_*e*_. These values were related to PAC in a correlation analysis to test the specific hypothesis of a functional relationship between PAC and behavioral performance (see Section 2.7).

### 2.7. Frequency analysis

In the first step we analysed whether oscillations show significant amplitude variations following the stimulus or the motor response. The rationale was to exclude the possibility that expected effects of PAC could be the result of the variation of only one frequency. We filtered the epochs in each trial in a broad range of frequencies ranging from 4 to 150 Hz (center frequencies) with a step of 2 Hz (band width of 4 Hz). By means of the absolute Hilbert transform we estimated the envelope of the oscillatory activity for each filtered time series in each trial. We then grouped the trials according to the HCC and LCC condition (see Section: Cognitive Control). In each frequency band and at each time point we compared the amplitude values across subjects and trials with a *t*-test. To assess statistical significance we corrected the significance threshold with a false discovery rate (FDR). Therefore, we fitted a cumulative normal distribution function to all *p-values* < the uncorrected significance threshold (*p* < 0.05; see Figure 4). All comparisons between HCC and LCC whose *p-values* < 0.05 in this new distribution were considered statistically significant. We furthermore asked, whether the amplitude in experimental conditions evolves differently with respect to their baseline. In each trial we calculated the differences between the baseline (average of 500 ms before motor response) and the averaged activity in the temporal interval of 500 ms following the motor response. We then tested by means of a *t*-test for differences between the experimental conditions (HCC-LCC).

### 2.8. Phase-amplitude cross-frequency coupling (PAC)

#### 2.8.1. Calculation of PAC

**Figure 2:**
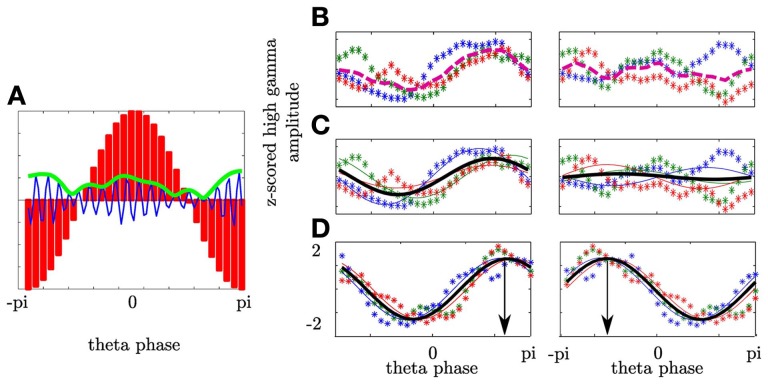
**Phase-amplitude cross-frequency coupling describes the dependency of high frequency amplitude on phase of low frequency oscillation. (A)** θ oscillation was separated into 30 phase bins covering the entire cycle (-π to π; red bars). In each bin the magnitude of the high γ analytic amplitude (green line) of the ongoing high γ band (blue line) was calculated. **(B)** Depiction of high γ amplitude as a function of θ phase for three subjects (colored asterisks) in one temporal interval. For each subject the mean high γ amplitude corresponding to each of the θ phase bins was calculated across trials. Afterwards, the variance across the mean over subjects (dashed line) was calculated. In the left plot (contralateral NAcc) the variance of the high frequency amplitude across the θ bins is greater as compared to the variance across subjects for a given bin resulting in an enhanced averaged variance as compared to the ipsilateral NAcc (right plot). **(C)** The variation of high γ amplitude as a function of θ phase was predicted by a cosine function assuming a unimodal dependency of high γ amplitude on the θ phase. A cosine function was fitted to individual high γ amplitudes (solid lines) and pooled across subjects (bold black line). In the left plot a high modulation index results from higher variation across the θ cycle than amplitude differences between subjects. **(D)** PAC defines the θ phase the high γ amplitude is coupled to. Both plots show the same coupling strength, however, with different coupling phases. In the left plot the high γ amplitude is coupled to the descending part of the θ cycle whereas in the right plot the coupling phase is the ascending part of the θ cycle.

To define whether frequencies interact and whether this interaction shows a temporal pattern, we quantified the relationship between the phase of the θ frequency band and the amplitude of a high frequency band in a manner comparable to the approach of Tort et al. (2009). Specifically, for a given electrode *e* we used the temporal interval *i* around the button press in the fixed and random sequence trials (*N*_trials_ = 300) since in both the subject had to respond according to an external cue. In this interval *i* we separated the θ oscillation into 30 phase bins (−π to π; *N*_bins_ = 30) and calculated the averaged high frequency amplitude within each phase bin (see Figure 2A). We filtered the raw signal in the θ (4–8 Hz (Axmacher et al., 2010)) band and a high frequency band covering the γ and high γ bands. The high frequency band was divided into narrow sub-bands with center frequencies ranging from 25 to 175 Hz, a bandwidth of 30 Hz and a step size of 2 Hz. We used the Hilbert transform to estimate the high frequency amplitude time series and the θ phase time series. From this the amplitude-phase-histograms were derived with the 2π−θ-cycle split into 30 phase bins of equal width (0.21 rad or 12°) in consecutive temporal intervals. We first calculated the cross-frequency spectrogram (amplitude variation of a high frequency oscillations as a function of the phase of a low frequency oscillation) within each subject. This means that the θ-phase values over the temporal interval (166 ms) were sorted into 30 equally spaced phase bins. The window size of each temporal interval was set such that a full cycle of the center frequency (6 Hz) of the θ range (4–8 Hz) was covered (166 ms). Next, the high gamma amplitudes observed at the various time points were separately averaged for each phase bin. As the next step in each subject we fitted a cosine function to the resulting high gamma amplitude values over phase bins. To prove the dependency of the high gamma amplitude on a same θ phase across subjects we averaged the resulting fit functions (see Figure 2). As the final step in our analysis we statistically compared whether more variance is explained by the variance across subjects or more variance is explained by the averaged fit functions (see next paragraph). If the latter was the case then all subjects' high gamma amplitude depended on the same θ phase and the Modulation Index was high. For example, if coupling did not rely on the same θ phase then the variance across subjects would be higher compared to the variance across the θ cycle. In sum, first the subject specific electrophysiology was evaluated followed by the statistics within the entire group of subjects in which we tested whether despite averaging the fit function more variance is explained by the fit function than by the variability across subjects. The same analysis was also conducted with 2 underlying θ cycles. These analyses yielded roughly the same results, but with a poorer temporal resolution. The window of analysis was shifted in time by 10 ms between −600 and 600 ms around both the stimulus and the motor responses. This led to 121 temporal intervals (N_interval_). Subsequently, in each interval the phase-amplitude distribution (distribution of high gamma amplitude values across theta phase-bins) was averaged across the electrodes separately for each hemisphere (contra- and ipsilateral to the performing hand) and for stimulus and response alignment.

#### 2.8.2. Quantification of PAC

To quantify PAC we used the variance σ^2^_PAC_ of the mean high frequency amplitude and the Modulation Index (MI; see Figures 2B,C). σ^2^_PAC_ is suited to be an exploratory measure since no specific model is assumed. In contrast the MI assumes a specific form of dependency of high frequency amplitude on the θ cycle and is defined by the strength of coupling and the phase to which the high frequency amplitude is coupled. In each temporal interval *i* we calculated the high γ amplitude distribution across the θ cycle per subject. We then averaged the high frequency amplitude across participants. This averaged high frequency amplitude is shown in Figure 2B as a dashed line. We calculated the variance σ^2^ of the averaged high frequency amplitude as a function of the θ phase

(2) $$\sigma_{\left(\bar{H\gamma /\theta }\right)}^{2}=\frac{1}{N_{\text{bins}}}\sum_{i=1}^{N_{\text{bins}}}{\left(a_{i}-\bar{a}\right)}^{2}$$

with *a*_*i*_ representing the mean high γ amplitude for a given phase bin across participants and *ā* representing the averaged amplitude of *a*_*i*_ over θ phases. The larger σ^2^ the larger are the differences of the high frequency amplitude at different θ phases. A high value of variance indicates a high concordance of PAC across participants. In Figure 2B we show this for two cases. The left plot shows a high variance of the averaged high frequency amplitude. The right plot shows that individual fluctuations of the high frequency amplitude are canceled out in the average. This leads to a small variance indicating a lack of coupling. To compare across different high frequency bands we normalized the amplitude values by *z-scoring*. We used the variance to compare the modulation of high frequencies by θ phase across all anatomical regions. Note that a high σ^2^ only indicates that at different θ phases the high frequencies differ in amplitude: it does not explain whether the variance of the high frequency amplitudes is accounted for the θ cycle. Therefore, in each high frequency showing a significant variance level we determined the goodness of a cosine fit (*F*-value). The cosine function (representing the θ cycle) was fitted to the *z*-scored high frequency amplitude values in each subject. The best cosine fit function minimizes the sum of squares of errors. We termed the test statistic *modulation index* according to Tort et al. (2009). However, in our analysis the MI represents an ANOVA and hence, specifies whether more of the variance in the high frequency amplitude (*MS*_cos_ - explained by the θ cycle) is explained by the variation across the θ cycle or across the participants (*MS*_error_—unexplained by the θ cycle). Therefore, we averaged the cosine fit functions across subjects and assessed whether despite averaging more variance of the high frequency amplitude is explained by the θ cycle than the variance across subjects.

The variance between the θ phases is given as

(3) $$MS_{\text{cos}}=\frac{SS_{\text{cos}}}{df_{\text{cos}}}$$

with *SS*_cos_ as the sum of squares of high frequency amplitude between θ phases and *df*_cos_ as the degrees of freedom. The variance within the θ phases is given as

(4) $$MS_{\text{error}}=\frac{SS_{\text{error}}}{df_{\text{error}}}$$

with *SS*_error_ as the sum of squares of high frequency amplitude within θ phases and *df*_error_ as the degrees of freedom. *MS*_error_ takes the variability across subjects into account. The MI is given as the ratio between both as

(5) $$MI=\frac{MS_{\text{cos}}}{MS_{\text{error}}}$$

The larger the MI more of the variance in the high frequency amplitude is explained by the variation across the θ cycle than across the participants. In fact this MI is comparable to an ANOVA which directly compares an effect of a condition (here θ cycle) in relation to a random variable (here individual high γ amplitude values) in each factor of the condition (here each single phase bin of the θ cycle). If each subject would show coupling however, with a strong coupling phase angle shift this would result in a low MI. Also strong gamma bursts in one subject and hence not a smooth variation of the high gamma amplitude across the θ cycle would result in a low MI since the variation across subjects increases compared to the variation across the θ cycle.

Furthermore, PAC is defined not only by the coupling strength but also by the phase of the θ cycle at which the high gamma amplitude reaches its maximum. In Figure 2D we show differences in coupling phase with the same coupling strength. Here all subjects show strong coupling since the variation of high gamma amplitude values in each subject is accounted for the θ cycle. The bold line shows the average of the individual cosine fit functions. Despite averaging across subjects more variance is accounted for the θ cycle than for variance across subjects at each phase. In the left plot high gamma amplitude is coupled to the descending part of the θ cycle whereas in the right plot the high gamma amplitude is coupled to the ascending part of the cycle. The coupling phase was estimated by determining the θ phase where the averaged cosine fit function reaches its maximum.

#### 2.8.3. Statistical test of PAC

To estimate the empirical distribution of σ^2^ in each temporal interval we calculated the variance of original time series filtered in the high γ range but randomly shifted in time as a function of the original θ phase in 500 randomizations. The 97.5th percentile of this distribution was used as the critical value when appraising the significance of our results. To estimate the empirical distribution of MI in each temporal interval we calculated the MI on the same set of randomizations as used for σ^2^. The 97.5th percentile of this distribution was used as the critical value when appraising the significance of our results.

#### 2.8.4. Functional relation between PAC and behavioral performance

We tested the specific hypothesis that a functional relationship between PAC and cognitive control exists. To this end we assessed the correlation between both RTs and *p*_*e*_ with PAC. In sliding windows of 50 consecutive trials with a step of 2 trials we calculated the grand average of reaction times (1. average of RT over trials, 2. average across subjects) and the *p*_*e*_. In separate analyses we tested both behavioral measures *p*_*e*_ and RTs for possible correlations with MI and coupling phase.

### 2.9. Preclusion of θ phase resetting

In each subject we investigated whether the coupling can be attributed to a resetting of the θ phase. At each time point across trials in each subject we calculated the phase concentration κ of circular data which is the reciprocal to the variance in a normal distribution to exclude the possibility that PAC results from phase realignment of the θ oscillation across trials. A high κ-value indicates a preferred θ phase across trials at a given time point. Statistical significance was assessed by a permutation procedure. In 500 runs the trial-wise time series were shifted in time separately. In each run κ was calculated. The confidence intervals for phase concentration κ were derived from the resulting 500 κ time series.

### 2.10. Specificity of θ-high γ coupling

We furthermore sought to preclude the possibility that the high frequency amplitude was coupled to the phase of frequencies other than θ. In the 200 ms following the motor response we calculated the variance of amplitude of all high frequency bands in the γ/high γ range (center frequencies: 55–165 Hz, bandwidth: 30 Hz, step size: 2 Hz) as a function of the phase of low frequencies ranging from 3 to 16 Hz (bandwidth: 4 Hz, step size: 1 Hz). Here, we first calculated the high frequency amplitude distribution across the cycle of each low frequency and then averaged across the subjects as shown in Figure 2. We then calculated the variance of the averaged high frequency amplitude distribution. The variance is high if all participants show a comparable distribution of the high frequency amplitude across the cycle of a given low frequency cycle. In contrast, the variance is low if participants did not show a comparable dependency of the high frequency amplitude on the low frequency cycle.

## 3. Results

### 3.1. Behavioral results

**Figure 3:**
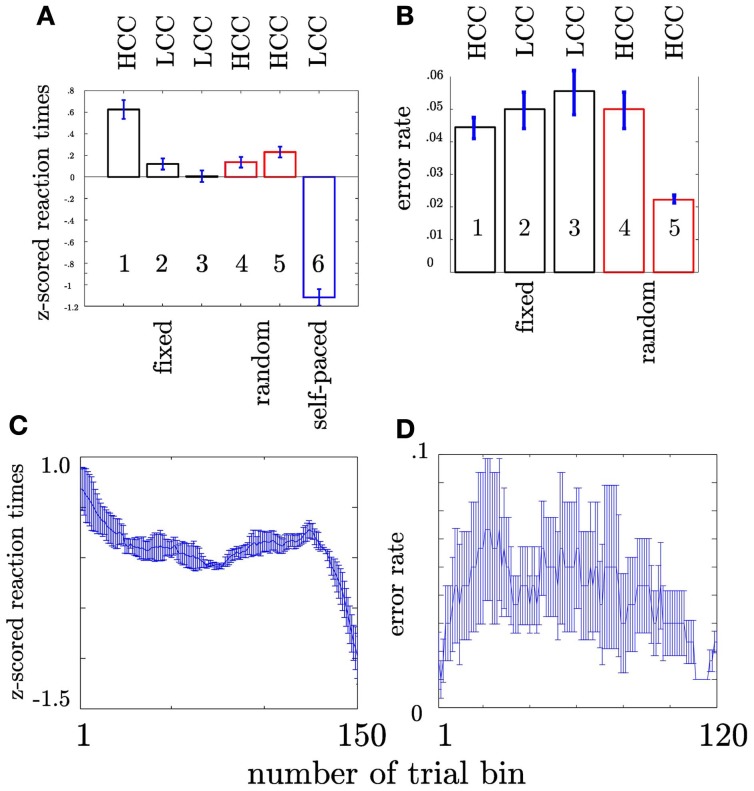
**Depiction of behavioral results. *x*-axis: sequence type**. Numbers within the bars show the number of the block. HCC and LCC above the bars indicate the level of cognitive control necessary to perform the task (HCC—high cognitive control and LCC—low cognitive control) **(A)** Reaction times were pooled across subjects and trials. Each bar encompasses reaction times of 180 trials (1 block). Error bars represent the standard error across trials. The color of the bar indicates the sequence type as in Figure 1. Trialwise reaction times vary in accordance with sequence type. For more clarity *post-hoc* differences are summarized in Table 2. Participants show increasingly faster response with training the fixed sequence. Following the fixed sequence reaction times are slower during the random sequence. **(B)** Error rates per block pooled across subjects. Error rates are only shown for fixed and random blocks since no error can be calculated for self-paced blocks. **(C)** Depiction of the evolution of RT across the trial bins. The error bars in both plots denote the standard error of the mean. **(D)** Depiction of the evolution of error rate across the trial bins. The error bars in both plots denote the standard error of the mean

First, we tested whether the participants performed differently throughout the task (see Materials and Methods) indicating differences in cognitive control. We assumed that tracking and responding to an unknown (i.e., block 1), a well-learned (i.e., block 2–3) or an unpredictable sequence (i.e., block 4–5) call for different cognitive control demands. Unknown and unpredictable sequences demand a high cognitive control since recent patterns cannot be extracted. Differences in reaction times across blocks (see Table 1) were confirmed with an ANOVA (*F*_(5, 1074)_ = 87.11, *p* < 0.0001; see Figure 3A). Figure 3C shows the mean reaction times for each trial bin. *Post-hoc* paired *t*-tests confirmed changes in reaction time between blocks. These results were summarized in Table 2. The relative comparison between blocks shows that blocks 2 and 4 show more similar mean RTs than blocks 1 and 4. However, it is of note that blocks 1 and 2 are statistically different as well and even though not statistically significant there is a trend from block 2 to 3 and all the more important from 3 to 4. Therefore, the overall trend across the experiments suggests that there is a course from HCC to LCC during the second and third block to HCC in both random sequence blocks and again to LCC in the self-paced block in terms of reaction times. In sum, we interpret the results in an absolute way (global course across the experiment) given that we recorded behavioral data from (i) non-healthy subjects which participated in (ii) only one block due to the limited recording time. Furthermore, blocks differed with respect to the error rate *p*_*e*_ which was confirmed with a χ^2^ test (mean *p*_*e*_ per block: 0.044, 0.050, 0.056, 0.050, 0.022, *p* < 0.005, please see Table A2 for the pairwise comparisons). Figure 3B shows the continuous increase of *p*_*e*_ from block 1 (HCC) to block 3 (LCC) and a decrease of *p*_*e*_ from LCC to HCC in block 4 and 5. Note, in the self-paced block 6 no errors could be made and hence *p*_*e*_ could not be calculated. In Figure 3D we show the error rate for each trial bin.

**Table 2:** ***Post-hoc* statistical *t*-tests**.

**Comparison**	***t*-value**	***p*-value**
1–2	5.03	<0.0001
1–3	6.09	<0.0001
1–4	4.91	<0.0001
1–5	3.96	<0.0001
1–6	15.09	<0.0001
2–3	1.52	0.13
2–4	−.22	0.82
2–5	−1.54	0.12
2–6	13.41	<0.0001
3–4	−1.8	0.06
3–5	−3.05	0.002
3–6	12.04	<0.0001
4–5	−1.33	0.19
4–6	13.73	<0.0001
5–6	14.72	<0.0001

The left column shows which blocks were tested. The middle column gives the **t**-value for significance of mean differences and the right column the corresponding **p**-value.

### 3.2. Amplitude variation

**Figure 4:**
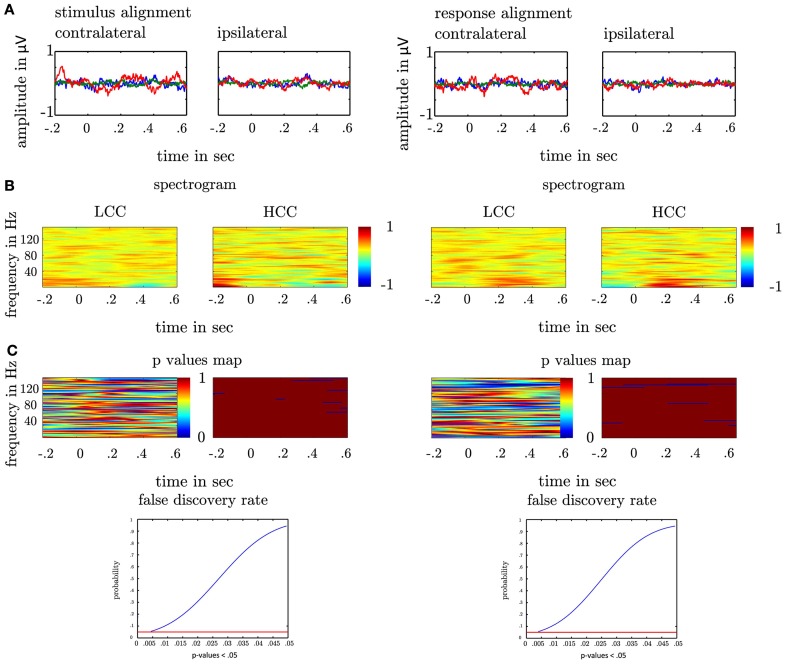
**(A)** Depiction of local field potentials locked to the response and the stimulus presentation both for the contra- and ipsilateral recording sites. The LFP in each trial was filtered between 3 and 200 Hz. Each colored line represents one subject. **(B)** Depiction of spectrograms for the low cognitive control condition (left-LCC) and the high cognitive control condition (right-HCC). In each subject we estimated the spectrogram by means of the Hilbert transform. In each trial aligned to the motor response/stimulus, we calculated the envelope of the bandpass filtered time series (4–150 Hz, bandwidth: 4 Hz, step: 2 Hz). The upper plots show the average across subjects and trials both aligned to the response and the stimulus in the HCC and LCC. For a better comparison of the amplitude across frequencies the spectrogram was *z*-scored for depiction only. All analysis were conducted on the non-standardized time series. The colorbar denotes the strength of amplitude in μV (*z*-score). **(C)** depicts the statistical evaluation by means of a *t*-test. At each time point in each frequency we calculated the *p*-value for the difference between LCC and HCC across subjects and trials (left plot). The right plot shows the time points and frequencies with a *p*-values < 0.05 (blue) on an uncorrected significance threshold. We corrected for multiple comparisons by taking the distribution of *p*-values < 0.05 into account. The 0.05% confidence interval of this new distribution (last row) served as a new significance threshold. Note that no *p*-value fell below this threshold indicating that between the HCC and the LCC condition no significant difference in amplitude modulation was found.

To exclude the possibility that PAC results can solely result from significant amplitude variations in one frequency we compared amplitude variations in a broad range of frequencies ranging from θ to high γ, which neither for the amplitude variation at each time point nor the test for different evolution with respect to the baseline passed the significance threshold FDR-corrected for multiple comparisons (see Figure 4).

### 3.3. High γ amplitude varies as a function of θ phase in the contralateral NAcc

**Figure 5:**
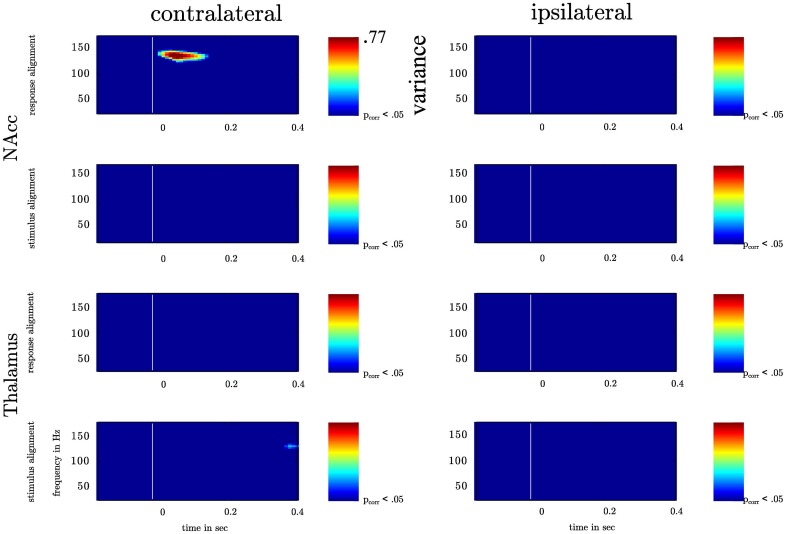
**Depiction of the variation of high γ amplitude across θ cycle for bilateral NAcc and bilateral anterior Thalamus**. The recorded time series in the contra- and ipsilateral NAcc and Thalamus were aligned to the motor response or the instructive stimulus. In each frequency band ranging from 25 to 175 Hz (centerfrequencies) we calculated the variance of high frequency amplitudes across the θ cycle (4–8 Hz) as a function of time. Variance increased significantly solely following the motor response in the contralateral NAcc (*p*-value corrected for the number of recording sites). Variance values corresponding with a *p*-value smaller than *p*_corr_ are depicted color-coded ranging from blue (small variance) to red (high variance).

**Figure 6:**
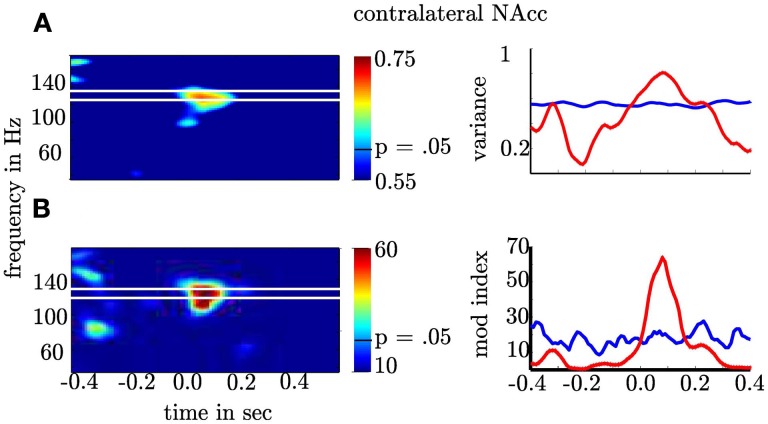
**Depiction of θ-high γ PAC for the contralateral NAcc. (A)** The first row gives the $\sigma_{\left(\bar{H\gamma /\theta }\right)}^{2}$ as a function of time (*x*-axis) and frequency (*y*-axis). **(B)** The second row shows the modulation index. The second column shows the frequency band (≈100–140 Hz; red line) with significant increase of $\sigma_{\left(\bar{H\gamma /\theta }\right)}^{2}$ and PAC. The blue lines denote the 97.5th percentile used as confidence interval of the normal distribution for σ^2^ and *F*-distribution for MI. Note that the variance and the Modulation index exceeded the significance threshold for more than 200 ms and each point is the result of a temporal interval of 166 ms. This means that coupling is present in non-overlapping temporal intervals and hence, extents across several cycles.

Our general hypothesis was that the high γ amplitude varies as a function of θ phase in the NAcc. We tested the statistical significance of σ^2^_PAC_ in the bilateral NAcc and the bilateral ANT associated both with the numeric stimulus and the response. We found an increase in σ^2^_PAC_ shortly after the motor response solely in the contralateral NAcc (see Figure 5) but not in the ipsilateral NAcc nor in the ANT. Figure 6 specifically shows σ^2^_PAC_ and MI for the contralateral NAcc. The increase in the contralateral NAcc was statistically significant, exceeding the 97.5th percentile of our computed distribution of gamma variances σ^2^_PAC_ (CI_97.5_ = 0.57; Figure 6A). By calculating the MI we then tested whether the variance of the high frequency amplitude is accounted for the θ cycle (see Figure 6A). As for σ^2^_PAC_ we found an increased MI following the motor response. The increase in the contralateral NAcc was statistically significant, exceeding the 97.5th percentile of our computed distribution of MI_random_ (CI_97.5_ = 18, see Figure 6A). The increased σ^2^_PAC_ of high gamma (100–140 Hz) amplitude in the contralateral NAcc could be accounted for the θ phase. This coupling was absent for the ipsilateral NAcc and following the stimulus, as well. We assumed that this is a result of different reaction times between subjects [*F*_(2, 1077)_ = 136.54, *p* < 0.0001]. We furthermore verified that coupling was restricted to the θ-high γ interaction (see Figure 12) and that an increase of coupling could be found in each subject (see Figure 13). The individual coupling patterns all show a different temporal layout following the coupling on the population level. However, only in the temporal interval of coupling on the population level in all subjects the MI tends to increase.

### 3.4. Coupling strength reflects cognitive control

**Figure 7:**
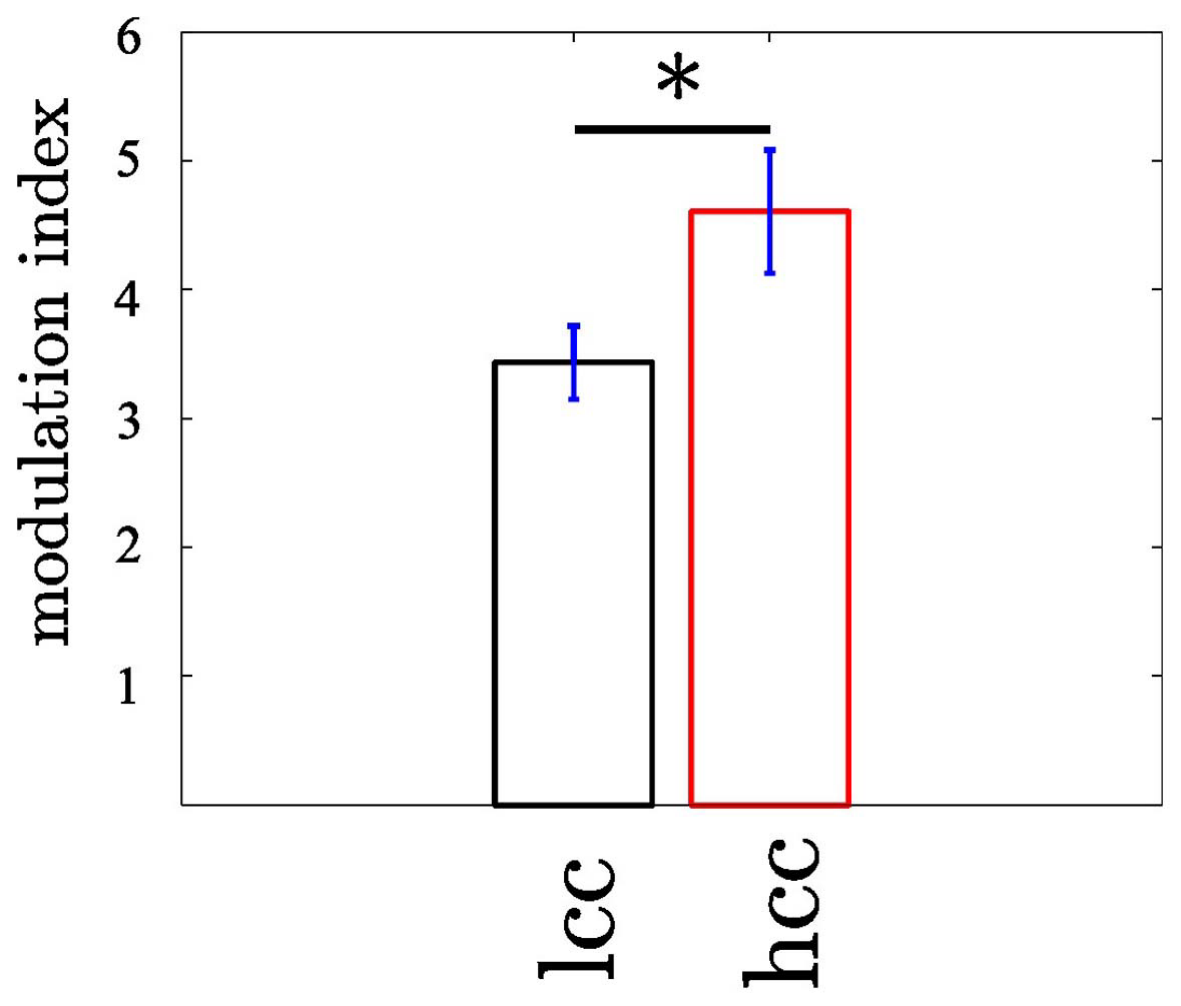
**The modulation of the high γ frequency by the θ phase differs between trials with low (LCC) vs. high (HCC) cognitive control (*p* < 0.05)**. The modulation index was calculated in non-overlapping trial bins of 10 trials. In trial bins containing only trials from the HCC condition the modulation was enhanced compared to trial bins of LCC trials. Error bars denote the standard error across trial bins. ^*^p < 0.05.

**Figure 8:**
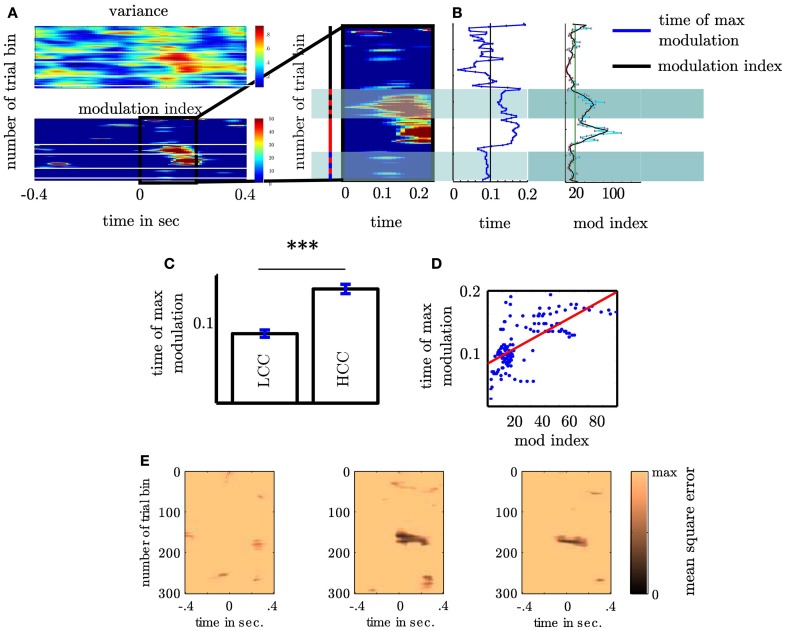
***Coupling strength varies with sequence type* (A)** Variance of high γ as a function of θ phase (upper plot) and modulation of high γ by θ phase (lower plot). Both plots show the variance and the modulation as a function of time (*x*-axis) and trial bin (*y*-axis). Trial bins are arranged from top to bottom. Both show that across subjects coupling is elevated at the beginning of the fixed and during the random trials. For consecutive trialbins each containing 50 trials we calculated the modulation index. Following the motor response the MI varied with sequence type. The sequence type is indicated by the colored bar left to the magnification: black corresponds to fixed, red to random and blue to self paced trials. The alternating red-black bar denotes those trialbins containing both fixed and random trials. Note that including only a small proportion of random trials elevates the modulation index. Alternating blue-black bar denotes those trialbins containing both random and self-paced trials. **(B)** Depiction of points in time at which modulation peaks for trialbins. The right panel shows the dependency of maximal modulation index on trial bin in the 0–200 ms interval (see Figure 5 right column) following the motor response. **(C)** In fixed trials coupling is earlier then in random trials. Only trial bins containing either fixed or random trials were used in this analysis. **(D)** Time of coupling between θ and high γ depends on coupling strength. **(E)** shows the individual summed square errors of each subject from the mutual cosine fit function as a function of trial bin. The greater error of subject 1 does not mean that this subject does not exhibit a sine shaped dependency of the high gamma activity as shown in Figure 2C. As the blue line deviates more strongly from the black bold line compared to the red and green line the summed mean error is greater than for subjects 2 and 3. However, it shows a clear sine shaped dependency. This means that subject 1 either contributes less to the strength of the MI or probably attenuates the MI but this does not challenge the visual impression that the high gamma amplitude is coupled to a resembling phase as subject 2 and 3. ^***^p < 0.001.

Our second question was whether the MI changes as a function of cognitive control. We tested this for the MI between θ (4–8 Hz) and high gamma band (112–142 Hz) since both bands showed the strongest coupling increase collapsed across all trials. An initial comparison of the MI in HCC versus LCC trial bins (each containing 10 non-overlapping trials) revealed that MI is significantly greater in HCC trial bins (*t*_16_ = −2.54; *p* = 0.016, see Figure 7). In the next step we tested whether the MI shows a systematic variation as a function of the experimental condition (HCC: block 1, 4, and 5 and LCC: block 2, 3, and 6). Therefore, we calculated the MI for the contralateral NAcc for each temporal interval across trial bins. Trial bins contained 50 trials with a step size of 2. Thus, we calculated the MI first for the trials 1–50, then for trials 3–53, and continued until trial 300–349 (see Figure 8A). Between 0 and 200 ms we determined when the maximal MI occurred. In periods of high cognitive control (HCC) the MI exceeded the statistical significance threshold (see Figure 8B). Coupling decreased in the low cognitive control condition (late fixed trial bins and final self-paced movement block). Importantly, coupling peaks earlier during the LCC sequences (mean coupling time = 90 ms) compared to HCC sequences (mean coupling time = 140 ms, see Figure 8C; *t*_99_ = −8.27, *p* < 0.1–12). The point in time of maximal coupling and MI covaried significantly (*r* = 0.7; *p* < 0.0001 see Figure 8D). Note, that differences in MI cannot be explained by longer RTs since trials were aligned to the motor response.

### 3.5. Functional relation between PAC and behavior

#### 3.5.1. MI correlates with error rate

**Figure 9:**
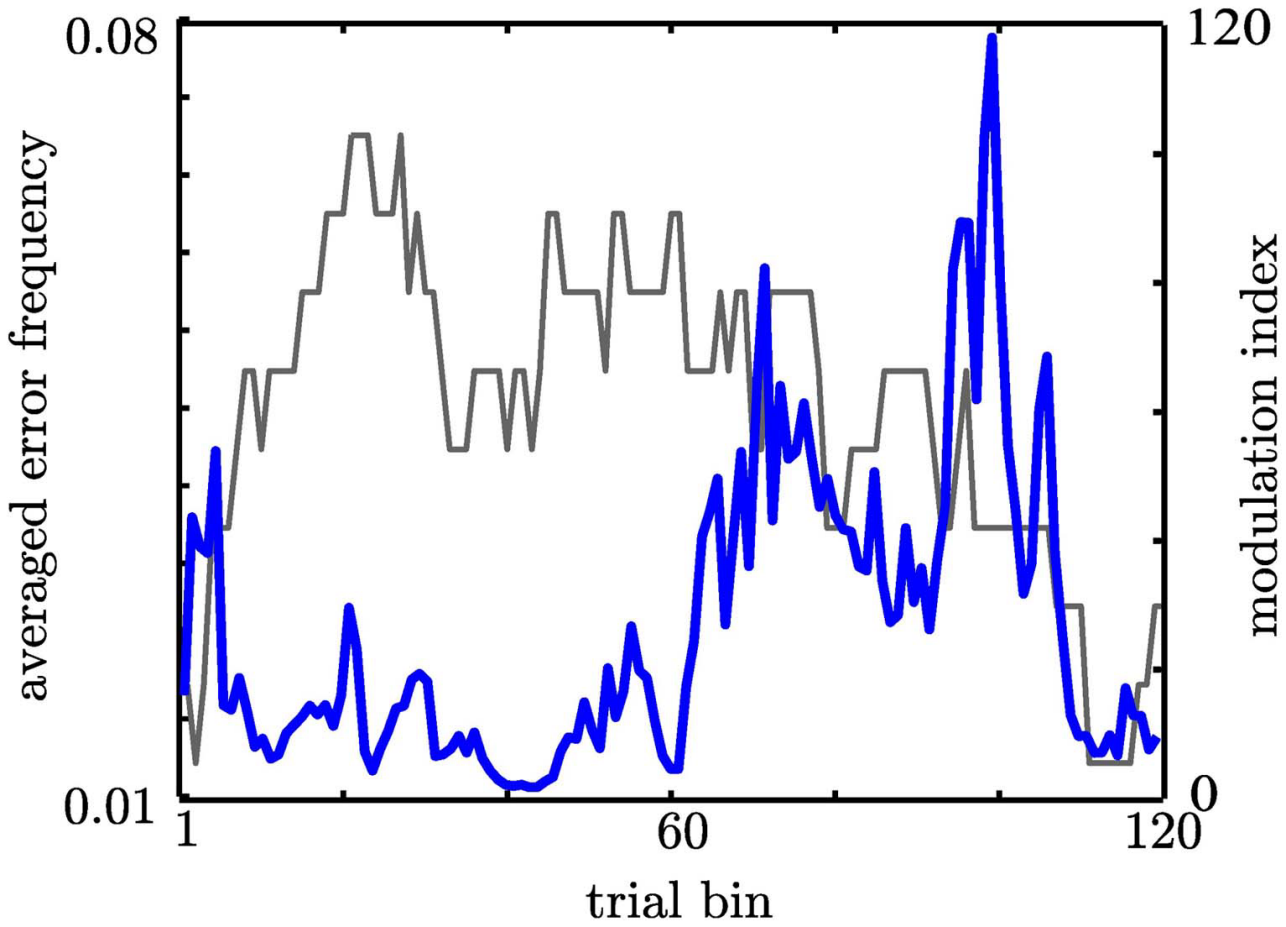
**Coupling strength predicts the probability to make an error as indicated by the correlation analysis**. Here we depict both correlated measures to illustrate the resembling course over time. The gray line gives the probability to make an error across subjects. The blue line shows the Modulation Index.

A further strong indication for functional relation between PAC and behavior would be provided by a covariation of MI with performance. It is assumed that the NAcc is engaged in action monitoring. We assume that high cognitive control allows for high action monitoring which should result in a low probability of making an error. In contrast, low cognitive control should result in a comparatively high probability of making an error. We therefore tested whether the PAC represented by the MI (coupling strength) or the coupling phase predicts the probability of making an error *p*_*e*_ or reaction times. To achieve a continuous measure in the consecutive trial bins containing 50 trials as described above we calculated *p*_*e*_. Subjects showed on average a reduced *p*_*e*_ at the beginning of the experiment and while performing the random trials where the modulation index was high, which accords with the view that action monitoring is high when subjects first begin to track the fixed sequences and during the random sequences (HCC). The MI was significantly correlated with *p*_*e*_ (*r* = −0.21; *p* < 0.05). Additionally, since the trial bins were not statistically independent we determined the significance of Pearson's correlation coefficient *r* against the distribution of *r* values calculated from 500 shuffles of trials. We found that the observed *r* value could not have been derived from a chance distribution (*p* = 0.01). This indicates a statistical significance. In Figure 9 we depict the *p*_*e*_ averaged across our three subjects (gray curve) together with the MI (blue curve). In contrast, MI did not vary with reaction times in the same trial bins (*r* = −0.15; *p* > 0.05).

#### 3.5.2. Coupling phase correlates with reaction times

**Figure 10:**
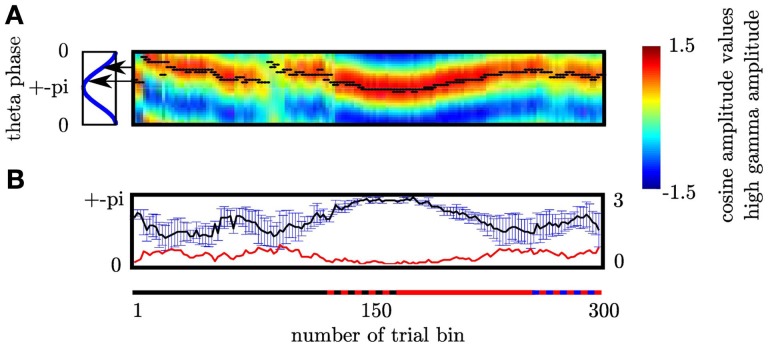
**(A)** Cognitive demands in different sequence types are reflected by difference in coupling phase. In each trialbin we determined the modulating phase of the θ cycle. Black dots denote the phase the high γ amplitude is coupled to for each trial bin. The sequence type is indicated by the colored line: black corresponds to fixed, red to random and blue to self paced trials. The alternating red-black line denotes those trial-bins containing both fixed and random trials. Alternating blue-black line denotes those trial-bins containing both random and self-paced trials. Phase differences were investigated comparing trial bins containing exclusively fixed and random trials, respectively (solid black and solid red intervals). Black arrows mark the mean θ phase. Coupling phases differ significantly between fixed and random trial bins. Please note that at the beginning of the entire experiment, when the sequence to be learned is unknown high gamma amplitude peaks at the same frequency as during the unpredictable random sequence. **(B)** During HCC high gamma amplitude is coupled to the θ trough across subjects while during LCC the coupling phase is different across subjects as indicated by the greater errorbars during trialbins of LCC. In each trial bin we grouped the high gamma amplitude according to the 30 θ bins for the temporal interval ranging from 0 to 200 ms. We fitted a cosine function to the resulting 30 high gamma amplitude values. The coupling phase of the θ oscillation was defined as the phase at which the high gamma amplitude was maximal. This leads to 3 coupling phases one per each subject in each trial bin. To better illustrate the in-phase coupling we collapsed the rising and the falling part of the θ cycle. Therefore, the y-axis ranges from 0 (θ peak) to ± π (θ trough). The x-axis gives the number of trial bins. The upper plot shows the average and the standard error of mean (SEM) of coupling phases across subjects for each trial bin. To better visualize this dependency of the SEM on the cognitive control we inserted the course of SEM over trial bins (red line) shows the standard error. This line demonstrates that the standard error across subjects is low in trial bins of HCC and high in trial bins of LCC.

Hasselmo et al. (2002) proposed a model of the functional relevance of the rat hippocampal θ rhythm in which the encoding and retrieval of memory information occur in different phases. He argues that this mechanism is important for the reversal of prior learning. Furthermore, we tested whether the coupling phase varies as a function of cognitive control. In each trial bin we determined the point in time of maximal MI, and evaluated the cosine function which served as the basis for the MI. The θ phase that corresponded with the maximal value of the cosine function representing the peak of the high γ amplitude was taken to be the coupling phase. We found the coupling phase discriminates between trial bins of HCC and LCC (see Figure 10). In trial bins containing fixed trials the mean θ phase is 1.72, while for random trials it is 2.29. A Watson–Williams test for circular data confirmed the significance of this coupling phase difference (F_(1, 99)_ = 23.6; *p* < 0.0001). Again, since the phase scores of each trial bin are not statistically independent, we calculated the significance of phase differences against the distribution of *F* values calculated from 500 shuffles of trials. We found that the observed *p* value could not have been derived from a chance distribution (*p* < 0.0001). As for the MI we tested whether the change of phase as a function of cognitive control has a significance for the observed behavior. We used ρ as the correlation coefficient between one circular and one linear random variable. In contrast to the MI we found the coupling phase is significantly correlated with the reaction times (ρ = 0.55, *p* < 0.001; see Figure 10A). ρ could not have been found within the set of 500 trial shuffles (*p* < 0.001). However, the coupling phase did not predict pe (ρ = 0.15, *p* > 0.05).

Note that there is a slight phase angle shift between the subjects. However, as revealed by the MI, this phase shift does not influence that more variance is explained by the variation across the theta cycle than between subjects. This is explicitly considered in our MI measure. In trial bins (see paragraph on Functional Relation between PAC and behavioral performance) of HCC (long reaction times and a low error rate as at the beginning of the experiment and during random sequence trials) subject's coupling phase is the same, namely around the trough of the θ oscillation (see Figure 10B). In contrast, during phases of LCC (during late trials of the fixed sequence and during self-paced sequence trials) subjects show a great variation of the coupling phase. This is indicated by the greater errorbars during trialbins of LCC (see Figure 10B). In each trial bin we grouped the high gamma amplitude according to the 30 θ phase bins for the temporal interval ranging from 0 to 200 ms. We fitted a cosine function to the resulting 30 high gamma amplitude values. The coupling phase of the θ oscillation was defined as the phase at which the high gamma amplitude was maximal. This leads to 3 coupling phases one per each subject in each trial bin. To better illustrate the in-phase coupling we collapsed the rising and the falling part of the θ cycle. Therefore, the *y*-axis in Figure 10B ranges from 0 (θ peak) to ± π (θ trough). The *x*-axis gives the number of trial bins. The upper plot in Figure 9B shows the average and the standard error (error bar) of coupling phases across subjects for each trial bin. The red line shows the standard error. This line demonstrates that the standard error across subjects is low in trial bins of HCC and high in trial bins of LCC.

### 3.6. θ phase re-alignment

**Figure 11:**
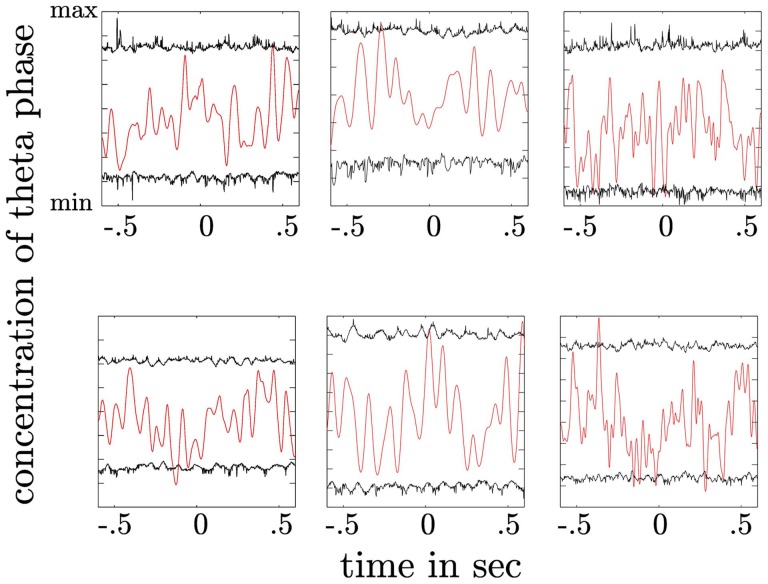
**Depiction of theta phase concentration as a function of time (red line) together with the confidence intervals derived from a permutation procedure (black lines)**. The upper plot shows the concentration parameter κ as a function of time for trials aligned to the stimulus. The lower plot shows the same for trials aligned to the response. High concentration values indicate a preferred θ phase across trials at a given time point. Concentration values exceeding the upper confidence interval would indicate a statistically significant alignment of the θ phase across trials. In both plots no significant θ phase alignment can be observed.

We tested for each subject whether PAC results can be a mere result of theta phase re-alignment. Here, we compared the phase concentration of the θ oscillation with an empirical distribution (see Materials and Methods). We did not find a statistically significant phase re-alignment in none of the subjects neither following the stimulus nor the motor response (see Figure 11).

### 3.7. Specificity of θ-high γ coupling

**Figure 12:**
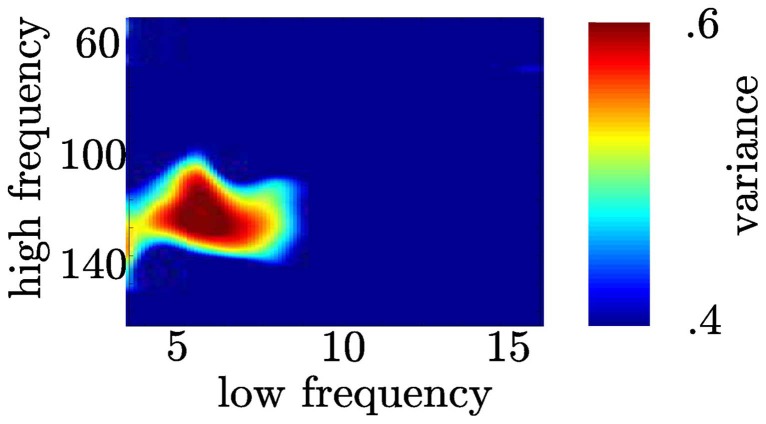
**Coupling following the motor response is restricted to the θ and high γ range**. In the time range between 0 and 200 ms (see A right column) we calculated coupling strength between narrow high frequency bands (centerfrequencies: 50 to 180 Hz, bandwidth: 30 Hz, step size: 2 Hz) and narrow low frequency bands (centerfrequencies: 3 to 16 Hz, bandwidth: 4 Hz, step size: 1 Hz).

We tested whether θ and high gamma activity exclusively show coupling or whether other frequency combinations also show coupling. We found the variance of the high frequency bands (≈100–140 Hz) across the phase of the θ (4–8 Hz) band was higher than any other frequency combination (see Figure 12). This yields a comparable narrow frequency interaction as found by Tort et al. (2008).

**Figure 13:**
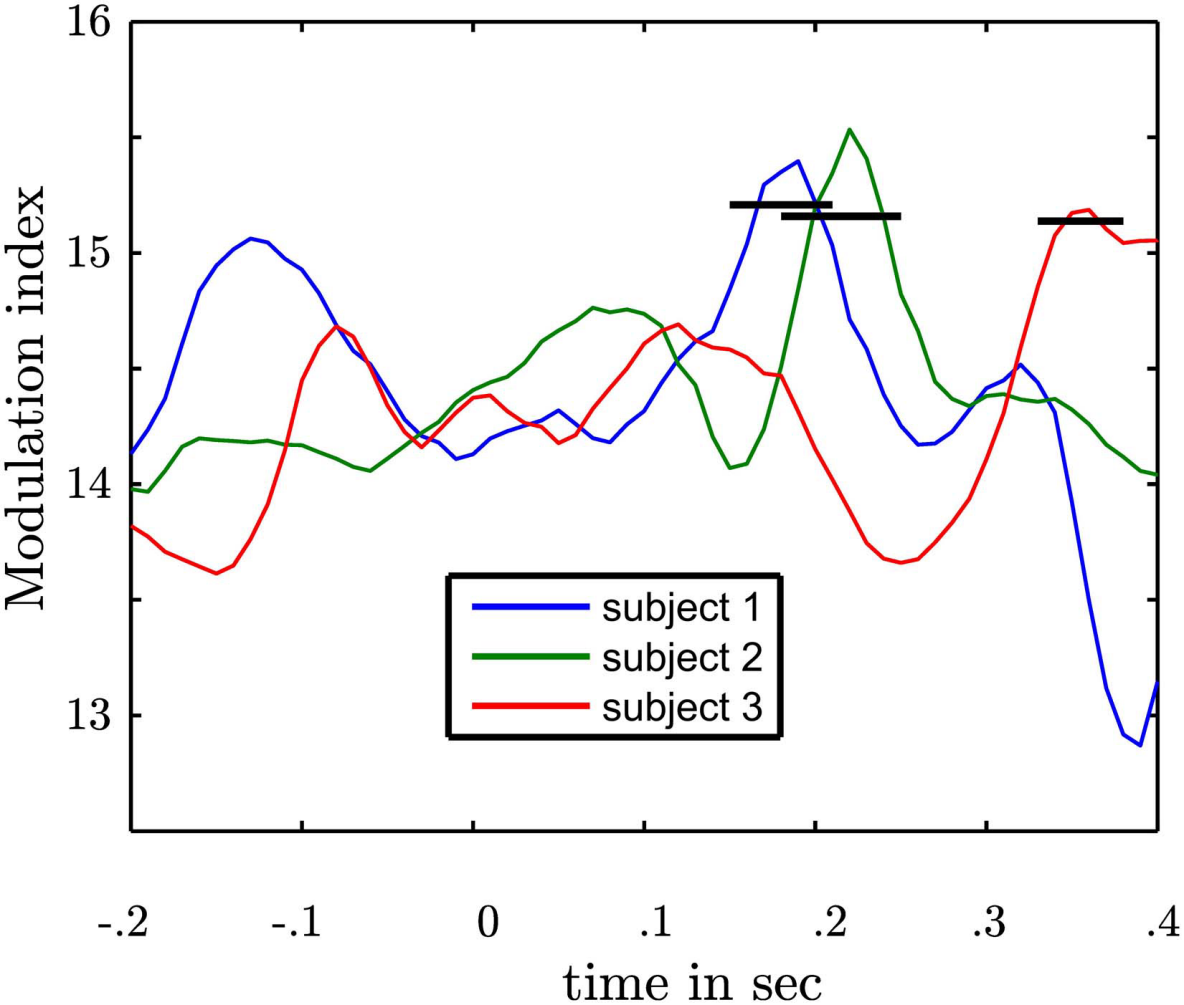
**Depiction of subject specific MIs in the contralateral NAcc following the motor response**. Each line represents the modulation index as a function of time of one subject. 0 marks the response time. Following the motor response in each subject an increase of modulation strength is observable. The black bars represent the upper confidence intervals derived from a permutation procedure in each subject. Following the motor response in each subject a statistically significant coupling was found.

## 4. Discussion

### 4.1. Summary of results

We investigated the dynamics of PAC in the human NAcc and show, that in the NAcc contralateral to a movement the θ phase modulates the high gamma amplitude (≈100–140 Hz) following a motor response. Importantly, this previously undescribed oscillatory pattern in the human NAcc increases with cognitive control and predicts behavioral adaptation as reflected in the reduction in error rates. Compared to reaction times the error rates show a more sluggish change which may explain the resemblance of the error rate if averaged across blocks. This means that changes in terms of RTs are more closely confined to the definition of blocks while the error rate changes with a greater time lag. However, the temporally resolved course revealed strong changes during the course of the experiment. We observed the strongest PAC in the first part of a task in which subjects responded to an unfamiliar fixed order stimulus sequence, and during responses to stimuli presented in a random order that required high load of cognitive control. In contrast, in periods with low cognitive control demands, i.e., when subjects responded to already learned stimulus sequences and during self-paced sequences, PAC was reduced. This pattern of response locked PAC cannot be accounted for reaction time differences since analyzed epochs were locked to the subject's responses, and no PAC was observed when epochs were locked to the stimulus presentations. Hence, coupling takes place in the temporal interval following a decision. This pattern is consistent with coupling patterns observed in rats by Tort et al. (2008). These investigators found enhanced coupling between θ and high gamma (80–120 Hz) within and between the striatum and the hippocampus. Coupling was strongest in epochs where a decision had to be made and thus were related to cognitive demands. Tort et al. (2008) hypothesized that PAC is a mechanism coordinating striatal and hippocampal learning circuits. As mentioned above, the hippocampus is strongly connected with the NAcc and a selective strengthening of this connection is assumed to be important for rapid facilitation of goal-directed behavior (Goto et al., 2005). The result that PAC decreases with learning indicates that PAC is related with the facilitated goal-directed behavior as proposed by Goto et al. (2005). That PAC occurs whenever a high level of cognitive control has to be applied supports the notion that PAC qualifies for facilitation. Thus, our data indicate that PAC can be a mechanism of information integration, since it occurs during high cognitive control supporting the hypothesis that PAC provides an effective mechanism to recruit local networks from functional related global networks to gate information (Buzsaki et al., 2004; Canolty et al., 2006).

### 4.2. Interplay of frequencies for integration of information

It is assumed that the control of motor behavior in NAcc is accompanied by release of dopamine (Wilson et al., 1983). Münte et al. (2007) found the human NAcc involved in error-related modulation which preceded scalp error-related negativity (ERN). On a behavioral level NAcc activity inhibits specific conditioned motor behavior (Wilson et al., 1983). In the present study the patients probability of making errors systematically varies during the course of the experiment. This variation matches with the course from HCC to LCC to HCC and again to LCC. We hypothesize that the variation of making errors signals a change in motor behavior and hence points to adaptation to the external demands as a result of learning. The error probability was especially low at the start of the experiment and during the random sequence task. The PAC in the contralateral human NAcc correlates with this change, which suggests that theta/high gamma coupling strength might be related with adaptation of behavior in later trials. This gains support by the PAC modulation occuring in early trials followed by behavioral adaptation. Once adapted in terms of reaction times and error rate no PAC modulation takes place. Thus, we speculate that the control for a specific action or motor routine as during an unknown or random sequence can be provided by enhanced coupling of θ-high gamma oscillations in the NAcc. This increased coupling becomes important when a subject has to switch from a previously established and automized motor routine to an unpredictable motor sequence. In contrast, a reduction in coupling, as observed during the tracking of the learned sequence, might facilitate automization. In our study enhanced action monitoring is especially important during learning of the fixed sequence and during the random sequence, since automatic behavior has to be interrupted. To achieve this, the NAcc has to integrate information between different cerebral units. This could be the information necessary to control the gating into the motor system from the limbic system and the prefrontal cortex (Mogenson et al., 1980). The NAcc neuronal activity is mutual depend on hippocampal input (O'Donnell et al., 1995) as well as PFC activity (French et al., 2002). Thus, the complex connections with limbic, prefrontal and motor structures make the NAcc an ideal site for the integration of information which is supported by the phase-amplitude cross-frequency coupling mechanism.

### 4.3. PAC is strongest following a decision

Tort et al. (2008) reports that in the rat's striatum and hippocampus PAC was strongest during the decision phase while rats navigated through a maze. In contrast, in our experiment the period of movement was short because there was only a single button press. This can explain differences in the timing of PAC patterns. In the present study, PAC occurred shortly after the decision was made and correlated with the probability of making an error, suggesting that changes in response selection based relative to past experiences calls for the coordination of information carried by different frequencies. Based on our results, we propose that the human NAcc signals the unpredictability of a future external event to which a response will have to be given, thereby indicating the necessity of stopping an automated response. This is accomplished by integrating information in the θ and high gamma band. In support of this contention Berns et al. (2001) found the reward system and especially the human NAcc responsive to different levels of predictability. In particular, the NAcc was more active during an unpredictable sequence, in line with our finding that PAC was elevated at the beginning of learning and during the tracking of the random sequence. Further, O'Donnell et al. (1995) found differences in activity in the striatum, especially in the putamen and the nucleus caudatus adjacent to the NAcc, for automated vs. unfamiliar motor behaviors. The increased coupling following a decision under a condition of high cognitive control might represent a type of associative memory which combines information about events (button press) and the context (stimulus presentation) (Tort et al., 2009). Here one can image that the association which is acquired more easily under higher cognitive control provides the subject with the possibility to respond faster. Furthermore, we observed two patterns of the course of coupling. In the first, coupling decreased during fixed trials and in the second coupling increased during random trials and both differ with respect to the predictability of the upcoming event and hence which finger to move. This says that during fixed trials the subjects are informed based on the memory of past trials which finger is to move and hence the finger movement options are limited to one finger. In contrast in the random trials the subject has to hold up three finger movement options (3 since 4 different stimuli are presented with the constraint that no stimulus was consecutively repeated twice). This might also explain why PAC increases constantly during random trials. Alternatively, this pattern can be a result of the monitoring of the recent action which is underscored by the temporal relation. This interpretation is supported by the involvement of the human NAcc in action monitoring—the error detection and correction (Münte et al., 2007). The correlation of the modulation strength with the error rate indicates that NAcc activity is involved in action monitoring. Action monitoring in turn involves a comparison between the representations of an appropriate response and the response actually made (Scheffers and Coles, 2000). These diverge if a response error was committed. Error monitoring is accompanied by prominent scalp potentials (Nieuwenhuis et al., 2001) and the activity in the NAcc is involved in error-related modulations (Münte et al., 2007). The authors have shown that the early surface potential which indicated the error detection was preceded by NAcc activity. Accordingly, activity in the NAcc should contribute to ERN when the error rate is high. Moreover, the NAcc activity involved in error detection should occur earlier than the PAC modulation since the error signal on the scalp level is evoked around 100 ms and is delayed by the NAcc by about 40ms (Münte et al., 2007). Importantly, PAC modulation occurs when the error rate is small. This makes the PAC a complementary event to the error-related modulation. We speculate that PAC modulation occurs when the comparison process between the appropriate and the actual response revealed that no error was made. Therefore, PAC could be the signal involved in confirmation of the correct response which facilitates goal-directed behavior in later trials. Due to the vicinity to the motor response one could argue that PAC is a mere mechanical artifact, however coupling is observed only in one region and shows a tight functional correlation with behavioral measures. Furthermore, in case of an artificial result we expected coupling to be represented across a broad band of low coupling and high coupled frequencies. However, coupling was restricted to the θ – high gamma range. Hence, we precluded PAC to be a result of an artifact as a possible explanation. Based on this, we speculate that PAC in the NAcc signals a deviation from expectancy: a negative reinforcement that implies the need to stop an automated motor routine in which learned responses are pre-activated to reduce reaction times.

### 4.4. Differences in coupling phase

During the course of the experiment the coupling phase of θ oscillations varied systematically, with coupling close to the θ trough during early learning and tracking the random sequence (high cognitive control), and coupling to the descending part of the θ cycle during tracking a well-learned fixed sequence (low cognitive control). A comparable result was found in a study conducted by Belluscio et al. (2012). In this study high γ activity (90–150 Hz) peaked near the θ trough during running but was coupled to the peak of the θ oscillations during REM-sleep. The authors hypothesized that modulation of the high gamma band by the θ band is state dependent. Here, we show that coupling in the human NAcc is also state dependent. The average coupling phase varies as a function of cognitive control applied by the subjects and parallels the results of (Belluscio et al., 2012). This strengthens our hypothesis that PAC might provide a mechanism to integrate information. (Hasselmo et al., 2002) highlighted the functional importance of different phases in the θ cycle for memory with the descending phase necessary for retrieval of memory and the trough for encoding of new information. They state that encoding of new information is facilitated if θ activity shifts in phase to accelerate the process of encoding. In our study when new information has to be encoded and the motor response has to be adjusted due to the new environmental requirements we observe strong coupling of the high gamma activity to the θ trough. The epochs in which new information has to be encoded are the trials in the early part of the fixed sequence and the random sequence which differ from the trials late in the fixed sequence with respect to the possibility of memory retrieval. Retrieval is only possible when the fixed sequence has been learned distinguishing between the two distinct cognitive states.

## 5. Conclusion

Together these results show that motor learning is accompanied by a complex interplay of θ and high gamma activity. In the NAcc contralateral to the performing hand the coupling of these frequencies varies systematically with the experimental conditions which allowed the participants to perform differently.

## Funding

This study was supported by NIH Grant NS21135 (Robert T. Knight), DFG HE 1531/11-1 (Jurgen Voges, Hans-Jochen Heinze), DFG SFB 779 TP A11 (Hans-Jochen Heinze) and EU-Project ECHORD 231143 (Hermann Hinrichs).

### Conflict of interest statement

The authors declare that the research was conducted in the absence of any commercial or financial relationships that could be construed as a potential conflict of interest.

## Figures and Tables

**Figure A1:**
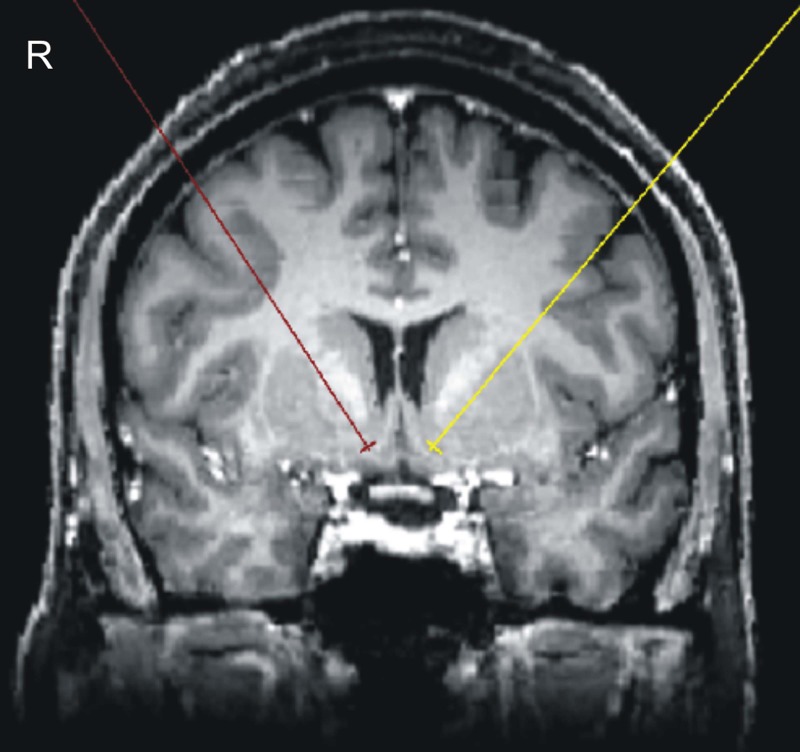
**Anatomic allocation of bilateral NAccs**.

## 6 Appendix

### 6.1 Procedure: surgery and deep brain stimulation

We performed a bilateral stereotactically guided implantation of quadripolar brain electrodes (model 3387, Medtronic, Minneapolis, MI, USA) in the Nucleus Accumbens (NAcc) and in the anterior nuclear group of the thalamus (ANT) of 3 patients for treatment of a longterm pharmacoresistant epilepsy. General anesthesia was employed during the surgery. The implantation was conducted due to clinical reasons and as part of the treatment of the epilepsy.

Treatment planning standards and the surgical procedure are described elsewhere in detail Voges et al. (2002). Briefly, the target for the deep brain stimulation electrode was defined using standard coordinates as the point 2 mm rostral to the anterior border of the anterior commissure at the level of the mid-sagittal plane, 3–4 mm ventral and 6–8 mm lateral of the midline (Mai et al., 2004), with these coordinates modified according to individual planning MRIs. An important landmark is the vertical limb of Broca diagonal band, which can be clearly visualized in coronal MRI-scans. The target was placed 2-0.5 mm lateral to this structure. Using a deep fronto-lateral approach, the two distal contacts of the DBS-electrode were placed in the caudo-medial accumbens, the third contact within the transition-area medial to the border of the abutting internal capsule, and the fourth highest contact in the most medial part of the capsule or in the transition area to the caudate. The contacts within the NAcc are placed in the caudo-medial part, which according to histochemical criteria represents the remnant of the shell area in the primate (Sturm et al., 2003). In contrast to rodents, in the primate the shell area has regressed and is no longer clearly distinguishable, except by the typical receptors that it carries.

In addition, electrodes were implanted in the bilateral anterior nuclei of thalamus (ANT) of all the patients. The anterior thalamic nucleus is located 5 mm lateral to and 12 mm above the midcommisural point according to the Schaltenbrand atlas. Since the anterior nucleus of the thalamus is readily visible on the floor of the lateral ventricle in MRI images, the exact location of this target could be directly specified in each patient. Intraoperatively the localization of the leads was documented by stereotactic x-ray imaging using x-ray tubes installed in the OR.

In addition, we performed postoperative CT-examination (2 mm slice thickness). After a transformation of postoperative CT- and X-ray images to align them with the stereotactic treatment planning-MRI, we defined the stereotactic coordinates of each electrode lead contact and visualized these anatomical positions on corresponding sections of two stereotactic brain atlases of (Morel et al., 2007). The localization of the electrodes is depicted in Figure A1. To further indicate the coordinates of the most caudal NAcc electrode, we transformed all patients MRI images to MNI-space. The resulting individual MNI coordinates were (x, y, z): Pat 01: left (−6.9 5.2 −11.9), right (4.3 5.4 −10.4); Pat 02: left (−8.6 3.5 −8.6), right (9.6 3.7 −9.6); Pat 03: left (−8.3 7.5 −11.0), right (−6.2 7.3 −11.0). Postoperatively the electrode leads were externalized to allow electrical test stimulation with different parameters and recording from the depth contacts during different psychological tasks. Finally the four electrode-leads were connected to a single impulse-generator (IPG; Activa-PC, Medtronic) placed subcutaneously beneath the right clavicle. To evaluate our hypotheses we analyzed data from the bilateral NAcc electrodes. To provide evidence of the specificity of our results for the NAcc we also performed a control analysis of the recordings from the bilateral thalamus. We found results described for the contralateral NAcc were not duplicated in the thalamus.

**Table A1:** **Patient Information**.

**Patient**	**Gender, age, duration of epilepsy**	**Epilepsy**					**IQ**	**Verbal memory**		**Figural memory**	**Sustained Attention**
		Syndrome	lateralization	seizure onset	etiology	AEDs	(HAWIE-R)	Immediate free recall (*z*-score)	Delayed free recall (*z*-score)	Recognition (*z*-score)	(*z*-score)	(*z*-score)
Pat 01	F, 52, 19	multifocal	bilateral	mesiotemporal	cryptogenic	LCM	74.2	40 (−0.65)	5 (−1.6)	9 (−1)	8 (−1.6)	199 (−2.2)
						400 mg						
						LTG						
						200 mg						
Pat 02	M, 35, 9	focal	right	temporal	right	LEV	124.9	57 (−0.65)	12 (0.2)	15 (1.1)	39 (−1)	486 (0)
					temporal	2000 mg						
					encephalocele	ESL						
						1200 mg						
Pat 03	F, 28, 12	multifocal	bilateral	temporal	cryptogenic	LTB	94	53 (−0.4)	13 (0.2)	15 (1.1)	23 (−1)	424 (−0.8)
						200 mg						
						LCM						
						200 mg						

F, female; M, male, AED, antiepileptic drugs; LCM, Lacosamide; OXC, Oxcarbazepine; LTG, Lamotrigine; LEV, Levetiracetam; ESL, Eslicarbazepine acetate summarizes demographical and clinical information about the subjects

**Table A2:** ***post-hoc* statistical analysis of the error rate**.

**comparison**	***p*-value**
1–2	0.81
1–3	0.64
1–4	0.81
1–5	0.25
2–3	0.82
2–4	1
2–5	0.17
3–4	0.82
3–5	0.11
4–5	0.17

*The p-values refer to the pairwise comparisons of frequency by means of the χ^2^ of the summed number of errors across subjects per block*.

## References

[B1] Axmacher N. Henseler M. M. Jensen O. Weinreich I. Elger C. E. Fell J. (2010). Cross-frequency coupling supports multi-item working memory in the human hippocampus. Proc. Natl. Acad. Sci. U.S.A. 107, 3228–3233 10.1073/pnas.0911531107 20133762 PMC2840289

[B2] Barcelo F. Escera C. Corral M. J. Periez J. A. (2006). Task switching and novelty processing activate a common neural network for cognitive control. J. Cogn. Neurosci. 18, 1734–1748 10.1162/jocn.2006.18.10.1734 17014377

[B3] Belluscio M. A. Mizuseki K. Schmidt R. Kempter R. Buzski G. (2012). Cross-frequency phase-phase coupling between θ and γ oscillations in the hippocampus. J. Neurosci. 32, 423–435 10.1523/JNEUROSCI.4122-11.2012 22238079 PMC3293373

[B4] Belujon P. Grace A. A. (2008). Critical role of the prefrontal cortex in the regulation of hippocampus-accumbens information flow. J. Neurosci. 28, 9797–9805 10.1523/JNEUROSCI.2200-08.2008 18815264 PMC2879013

[B5] Berns G. S. McClure S. M. Pagnoni G. Montague P. R. (2001). Predictability modulates human brain response to reward. J. Neurosci. 21, 2793–2798 11306631 10.1523/JNEUROSCI.21-08-02793.2001 PMC6762527

[B6] Buzsáki G. Draguhn A. (2004). Neuronal oscillations in cortical networks. Science 304, 1926–1929 10.1126/science.1099745 15218136

[B7] Canolty R. T. Edwards E. Dalal S. S. Soltani M. Nagarajan S. S. Kirsch H. E. (2006). High gamma power is phase-locked to theta oscillations in human neocortex. Science 313, 1626–1628 10.1126/science.1128115 16973878 PMC2628289

[B8] Canolty R. T. Ganguly K. Kennerley S. W. Cadieu C. F. Koepsell K. Wallis J. D. (2010). Oscillatory phase coupling coordinates anatomically dispersed functional cell assemblies. Proc. Natl. Acad. Sci. U.S.A. 107, 17356–17361 10.1073/pnas.1008306107 20855620 PMC2951408

[B9] Chrobak J. J. Buzsáki G. (1998). Gamma oscillations in the entorhinal cortex of the freely behaving rat. J. Neurosci. 18, 388–398 9412515 10.1523/JNEUROSCI.18-01-00388.1998 PMC6793397

[B10] Cohen M. X. Axmacher N. Lenartz D. Elger C. E. Sturm V. Schlaepfer T. E. (2009). Good vibrations: cross-frequency coupling in the human nucleus accumbens during reward processing. J. Cogn. Neurosci. 21, 875–889 10.1162/jocn.2009.21062 18702577

[B11] Finch D. M. (1996). Neurophysiology of converging synaptic inputs from the rat prefrontal cortex, amygdala, midline thalamus, and hippocampal formation onto single neurons of the caudate/putamen and nucleus accumbens. Hippocampus 6, 495–512 8953303 10.1002/(SICI)1098-1063(1996)6:5<495::AID-HIPO3>3.0.CO;2-I

[B12] Fitts P. M. Posner M. I. (1973). Human Performance, Chapter 2, Learning and Skilled Performance. London: Wadsworth Publishing Company, 8–25

[B13] French S. J. Totterdell S. (2002). Hippocampal and prefrontal cortical inputs monosynaptically converge with individual projection neurons of the nucleus accumbens. J. Comp. Neurol. 446, 151–165 10.1002/cne.10191 11932933

[B14] Goto Y. Grace A. A. (2005). Dopamine-dependent interactions between limbic and prefrontal cortical plasticity in the nucleus accumbens: disruption by cocaine sensitization. Neuron 47, 255–266 10.1016/j.neuron.2005.06.017 16039567

[B15] Goto Y. Grace A. A. (2008). Limbic and cortical information processing in the nucleus accumbens. Trends Neurosci. 31, 552–558 10.1016/j.tins.2008.08.002 18786735 PMC2884964

[B16] Grace A. A. (2000). Gating of information flow within the limbic system and the pathophysiology of schizophrenia. Brain Res. Brain Res. Rev. 31, 330–341 10.1016/S0165-0173(99)00049-1 10719160

[B17] Grace A. A. Floresco S. B. Goto Y. Lodge D. J. (2007). Regulation of firing of dopaminergic neurons and control of goal-directed behaviors. Trends Neurosci. 30, 220–227 10.1016/j.tins.2007.03.003 17400299

[B18] Hasselmo M. E. Bodeln C. Wyble B. P. (2002). A proposed function for hippocampal theta rhythm: separate phases of encoding and retrieval enhance reversal of prior learning. Neural Comput. 14, 793–817 10.1162/089976602317318965 11936962

[B19] Knopman D. Nissen M. J. (1991). Procedural learning is impaired in Huntington's disease: evidence from the serial reaction time task. Neuropsychologia 29, 245–254 10.1016/0028-3932(91)90085-M 1829141

[B20] MacDonald A. W. Cohen J. D. Stenger V. A. Carter C. S. (2000). Dissociating the role of the dorsolateral prefrontal and anterior cingulate cortex in cognitive control. Science 288, 1835–1838 10.1126/science.288.5472.1835 10846167

[B21] Mai J. K. Paxinos G. Voss T. (2004). Atlas of the Human Brain 2nd Edn. San Diego, CA: Academic Press Elsevier

[B22] Mogenson G. J. Jones D. L. Yim C. Y. (1980). From motivation to action: functional interface between the limbic system and the motor system. Prog. Neurobiol. 14, 69–97 6999537 10.1016/0301-0082(80)90018-0

[B23] Morel A. (2007). Stereotactic Atlas of the Human Thalamus and Basal Ganglia. New York, NY: Informia Health Care Inc. 10.3109/9781420016796

[B24] Münte T. F. Heldmann M. Hinrichs H. Marco-Pallares J. Krämer U. M. Sturm V. (2007). Nucleus accumbens is involved in human action monitoring: evidence from invasive electrophysiological recordings. Front. Hum. Neurosci. 1:11 10.3389/neuro.09.011.2007 18958225 PMC2525987

[B25] Nieuwenhuis S. Ridderinkhof K. R. Blom J. Band G. P. H. Kok A. (2001). Error-related brain potentials are differentially related to awareness of response errors: evidence from an antisaccade task Psychophysiology 38, 752–760 11577898

[B26] Nissen M. J. Bullemer P. (1987). Attentional requirements of learning: evidence from performance measures. Cogn. Psychol. 19, 1–32 10.1016/0010-0285(87)90002-8

[B27] Notebaert W. Houtman F. Opstal F. V. Gevers W. Fias W. Verguts T. (2009). Post-error slowing: an orienting account. Cognition 111, 275–279 10.1016/j.cognition.2009.02.002 19285310

[B28] O'Donnell P. Grace A. A. (1995). Synaptic interactions among excitatory afferents to nucleus accumbens neurons: hippocampal gating of prefrontal cortical input. J. Neurosci. 15(5 Pt 1), 3622–3639 7751934 10.1523/JNEUROSCI.15-05-03622.1995 PMC6578219

[B29] Poldrack R. A. Sabb F. W. Foerde K. Tom S. M. Asarnow R. F. Bookheimer S. Y. (2005). The neural correlates of motor skill automaticity. J. Neurosci. 25, 5356–5364 10.1523/JNEUROSCI.3880-04.2005 15930384 PMC6725010

[B30] Ridderinkhof K. R. Ullsperger M. Crone E. A. Nieuwenhuis S. (2004). The role of the medial frontal cortex in cognitive control Science 306:443 10.1126/science.1100301 15486290

[B31] Scheffers M. K. Coles M. G. H. (2000). Performance monitoring in a confusing world: error-related brain activity, judgments of response accuracy, and types of errors J. Exp. Psychol. 26, 141–151 10.1016/j.neuron.2005.02.028 10696610

[B32] Siapas A. G. Lubenov E. V. Wilson M. A. (2005). Prefrontal phase locking to hippocampal theta oscillations. Neuron 46, 141–151 15820700 10.1016/j.neuron.2005.02.028

[B33] Sirota A. Csicsvari J. Buhl D. Buzsáki G. (2003). Communication between neocortex and hippocampus during sleep in rodents. Proc. Natl. Acad. Sci. U.S.A. 100, 2065–2069 10.1073/pnas.0437938100 12576550 PMC149959

[B34] Staudigl T. Zaehle T. Voges J. Hanslmayr S. Esslinger C. Hinrichs H. (2012). Memory signals from the thalamus: early thalamocortical phase synchronization entrains gamma oscillations during long-term memory retrieval. Neuropsychologia 50, 3519–3527 10.1016/j.neuropsychologia.2012.08.023 22975190

[B35] Sturm V. Lenartz D. Koulousakis A. Treuer H. Herholz K. Klein J. C. (2003). The nucleus accumbens: a target for deep brain stimulation in obsessive-compulsive- and anxiety-disorders. J. Chem. Neuroanat. 26, 293–299 10.1016/j.jchemneu.2003.09.003 14729131

[B36] Tort A. B. L. Komorowski R. W. Manns J. R. Kopell N. J. Eichenbaum H. (2009). Theta-gamma coupling increases during the learning of item-context associations. Proc. Natl. Acad. Sci. U.S.A. 106, 20942–20947 10.1073/pnas.0911331106 19934062 PMC2791641

[B37] Tort A. B. L. Kramer M. A. Thorn C. Gibson D. J. Kubota Y. Graybiel A. M. (2008). Dynamic cross-frequency couplings of local field potential oscillations in rat striatum and hippocampus during performance of a T-maze task. Proc. Natl. Acad. Sci. U.S.A. 105, 20517–20522 10.1073/pnas.0810524105 19074268 PMC2629291

[B38] Voges J. Volkmann J. Allert N. Lehrke R. Koulousakis A. Freund H.-J. (2002). Bilateral high-frequency stimulation in the subthalamic nucleus for the treatment of Parkinson disease: correlation of therapeutic effect with anatomical electrode position. J. Neurosurg. 96, 269–279 10.3171/jns.2002.96.2.0269 11838801

[B39] Wilson W. J. (1983). Nucleus accumbens inhibits specific motor but not nonspecific classically conditioned responses. Brain Res. Bull. 10, 505–5156. 10.1016/0361-9230(83)90148-X 6860977

